# MetaGeneTack: ab initio detection of frameshifts in metagenomic sequences

**DOI:** 10.1093/bioinformatics/bts636

**Published:** 2012-11-04

**Authors:** Shiyuyun Tang, Ivan Antonov, Mark Borodovsky

**Affiliations:** ^1^School of Biology, ^2^School of Computational Science and Engineering, ^3^Center for Bioinformatics and Computational Genomics, Georgia Institute of Technology, Atlanta, GA 30332, USA, ^4^Department of Biological and Medical Physics, Moscow Institute of Physics and Technology, Dolgoprudny, Moscow Region, Russia and ^5^Joint Georgia Tech and Emory University Wallace H Coulter Department of Biomedical Engineering, Atlanta, GA 30332, USA

## Abstract

**Summary:** Frameshift (FS) prediction is important for analysis and biological interpretation of metagenomic sequences. Since a genomic context of a short metagenomic sequence is rarely known, there is not enough data available to estimate parameters of species-specific statistical models of protein-coding and non-coding regions. The challenge of ab initio FS detection is, therefore, two fold: (i) to find a way to infer necessary model parameters and (ii) to identify positions of frameshifts (if any). Here we describe a new tool, MetaGeneTack, which uses a heuristic method to estimate parameters of sequence models used in the FS detection algorithm. It is shown on multiple test sets that the MetaGeneTack FS detection performance is comparable or better than the one of earlier developed program FragGeneScan.

**Availability and implementation**: MetaGeneTack is available as a web server at http://exon.gatech.edu/GeneTack/cgi/metagenetack.cgi. Academic users can download a standalone version of the program from http://exon.gatech.edu/license_download.cgi.

**Contact:** borodovsky@gatech.edu

**Supplementary information**: Supplementary data are available at *Bioinformatics* online.

## 1 INTRODUCTION

Metagenomic sequences are obtained from environmental microbial communities. The short sequences are usually sequenced using next generation sequencing (NGS) platforms such as Roche 454 and Illumina or traditional Sanger sequencing. Each platform produces reads of different lengths and has different error models. 454 sequencing platforms produce reads of ∼450 nt; errors are usually insertions and deletions (indels) in homopolymer regions. Illumina platform currently generates sequences of length ∼150 nt with substitutions constituting almost all errors. Sanger sequencing produces reads that may contain both types of errors, and the read length is ∼900 nt. Before gene calling, quality control methods are used to reduce errors on raw reads (e.g. trimming the error-prone 20–25 nt in Illumina 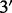 read ends). Short reads are assembled into longer (shotgun) sequences and contigs. In assembled sequences, the per-nucleotide error rate can be reduced from 0.5% in raw reads to as low as 0.005%. Still, errors may affect ∼3% to ∼4.5% of genes in assembled sequences (Luo *et al.*, 2012). Even several thousand nucleotides long metagenomic sequences do not carry enough sequence data to accurately estimate parameters of statistical models for protein-coding and non-coding regions. Moreover, the performance of conventional tools of gene prediction and annotation are impaired by indels in protein-coding regions (Hoff, 2009). On the other hand, tools of comparative genomics that have certain power in detection of frameshifts interrupting evolutionary conserved regions rely entirely on comparison with sequences from existing databases; these tools would not help to analyse novel genes and genes having low similarity with known genes (Kunin *et al.*, 2008).

Previously we have developed an algorithm and software program GeneTack (Antonov *et al.*, 2010), an ab initio tool for finding frameshifts in prokaryotic genomes. Since GeneTack requires a species-specific statistical model, it cannot work with sequences shorter than several hundred kilobases. The long enough sequence is necessary for self-training of GeneMarkS (Besemer *et al.*, 2001), the gene finder providing model parameters and gene predictions to GeneTack. Here we introduce an ab initio frameshift finder, MetaGeneTack, designed for metagenomic sequences. MetaGeneTack uses a heuristic method (Besemer *et al.*, 1999) to infer model parameters suitable for analysis of a short sequence at hand (e.g. 400 nt). A recently developed ab initio gene finder, FragGeneScan (Rho *et al.*, 2010), is also able to detect frameshifts in short sequences. We have assessed the performance of MetaGeneTack in tests on short sequences from 18 prokaryotic species. We have shown that MetaGeneTack performs comparably or better in frameshift detection than FragGeneScan.

## 2 MATERIALS AND METHODS

The idea of the heuristic method for building models of protein-coding regions is that frequencies of oligonucleotides, if cannot be derived directly owing to insufficient sequence length, can be inferred as functions of the sequence GC content. Thus, the oligonucleotide frequencies could be reconstructed as soon as we compute GC content of the short sequence that may serve as an estimate of GC content of the genome the sequence originated from. MetaGeneTack uses the fifth-order polynomial approximations of dependencies of hexamer frequencies on genome GC content derived from data on 582 annotated prokaryotic genomes (for details see Zhu *et al.*, 2010).

Heuristic method is used in two major steps of the MetaGeneTack pipeline. At the first step the pipeline is running MetaGeneMark (Zhu *et al.*, 2010), an ab initio gene finder using the heuristic method in the algorithm of analysis of short metagenomic sequences. This step identifies an initial set of protein-coding genes and selects a corresponding heuristic model (of bacterial or archaeal type of a particular GC content) for each sequence. The input sequence is then split into fragments where predicted genes are located in the same strand. The fragments assigned to the same model are grouped together. At the second step, the selected heuristic models are used in GeneTack for FS prediction in the grouped fragments.

To reduce the number of false positives, MetaGeneTack uses three post-processing filters for the initial FS predictions. Filter I rational is as follows. In a frameshifted gene with two overlapping protein-coding open reading frames (ORFs), the downstream ORF should not possess a functional ribosomal binding site (RBS). Therefore, if a gene predicted in the downstream ORF has a high RBS score (>2.0), the FS prediction is filtered out. Filter II is based on the following observation: in high GC genomes, true FS is separated by a rather long distance from a stop codon terminating the upstream ORF. Therefore, a predicted FS situated on a short distance from the stop codon (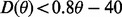, with *θ* designating GC content in percentage scale) is filtered out. Filter III works as follows: if an FS is predicted too close (<50 nt) to a border of the putative frameshifted gene or to 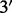 or 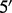 end of the sequence fragment, such FS prediction is filtered out. Filters II and III are applied to fragments with high GC content (θ > 50) and low GC content (θ ≤ 50), respectively. As a training set for assignment of the filters’ parameters, we used genomic sequences of *Escherichia coli*. To produce the program output, the final set of FS predictions is mapped back to the initial metagenomic sequences and combined with gene predictions of MetaGeneMark, thus producing the full list of genes with or without frameshifts.

To evaluate the accuracy of FS detection, we generated multiple test sets accounting for different sequence lengths and error models. To simulate metagenomic data, 18 prokaryotic genomes with GC content ranging from 28% to 75% were cut into 400 nt, 600 nt and 800 nt fragments (see Supplementary Table S1 for genome names). In a given genome, we selected 2000 fragments of each length. Frameshifts (indels) were similated in coding regions of a certain fraction of the fragment set: 5%, 10% and 20% of all fragments. Selection of the 400 nt as the minimum fragment length is in agreement with the conventional practice where fragments shorter than 400 nt are used for detecting nucleotide polymorphisms and short functional motifs (Wooley *et al.*, 2010). Selecting 5%, 10% and 20% fractions correspond to current estimates of per-nucleotide error rates in metagenomic fragments assembled from raw reads ranging from 0.0065% to 0.05%.

In the simulations, the indels were made in long stretches of coding regions (>200 nt) at a random location, separated by a distance of at least 50 nt from the fragment boundary. If an FS was predicted in the 20 nt vicinity of the true FS position, it was reported as a true positive, otherwise as a false positive.

## 3 RESULTS

**Fig. 1.**
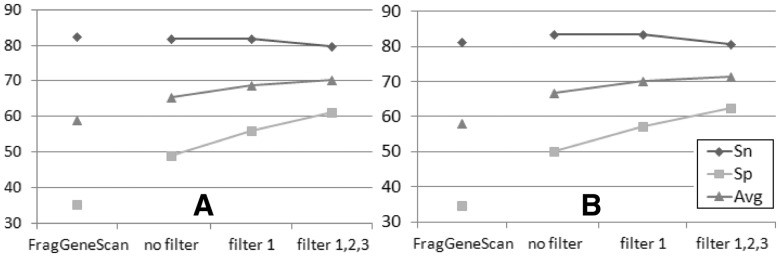
Performance of MetaGeneTack with different combinations of filters as well as performance of FragGeneScan (the leftmost columns) using the 600 nt sequences with 20% having simulated FSs as the test set. The predicted frameshift is reported as true positive if it is located within 20 nt from the true simulated frameshift position, (**A**) for fragments with insertions, (**B**) for fragments with deletions. Values are averaged among 18 genomes

Using *A* to denote the number of all FS predictions, *T* to denote the number of predicted true positives and *S* to denote the number of simulated frameshifts, we calculated sensitivity, 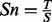 and specificity 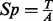. Note that this definition of specificity, accepted in bioinformatics literature, corresponds to the definition of precision in machine learning publications. Accuracy of MetaGeneTack was compared with accuracy of FragGeneScan (version 1.15, downloaded from http://omics.informatics.indiana.edu/FragGeneScan/). FragGeneScan requires users to select a sequencing method likely used for generating the input sequence along with indication of approximate sequencing error rate. We chose the option Sanger sequencing with 0.5%, as it yielded the best results of FragGeneScan among all available options. The *Sn* and *Sp* values averaged on the whole set of genomes are shown in Table 1. To give an example of genome specific values of *Sn* and *Sp,* we provide Supplementary Table S1 for the set of 400 nt fragments with 20% containing FSs. Results are averaged between sets of fragments with FS made by insertions and FS made by deletions (see also Fig. 1).

**Table 1.** FS detection accuracy of FragGeneScan and MetaGeneTack for short fragments from 18 prokaryotic genomes

Fragment length	Fragments having FS (%)	FragGeneScan			MetaGeneTack		
		*Sn*	*Sp*	Avg	*Sn*	*Sp*	Avg
400 nt	5	79.6	15.8	47.7	74.4	38.3	56.4
	10	80.5	27.3	53.9	75.3	54.5	64.9
	20	81.0	43.2	62.1	75.8	70.2	73.0
600 nt	5	81.2	11.7	46.4	79.9	27.7	53.8
	10	81.8	21.2	51.5	79.9	43.1	61.5
	20	81.9	35.1	58.5	80.1	61.7	70.9
800 nt	5	81.9	9.1	45.5	81.7	21.7	51.7
	10	82.6	16.9	49.7	81.2	35.0	58.1
	20	82.8	29.4	56.1	81.5	51.9	66.7

Values are averaged among genomes and then averaged between insertion and deletion FS sets (see Supplementary Table S1 for details)

In terms of (*Sn* + *Sp*)/2, MetaGeneTack performed better than FragGeneScan by 7–12%. The values of FragGeneScan *Sn* and *Sp* differed by ∼55 percentage points, whereas for MetaGeneTack, this gap was much smaller. The differences were likely due to different methods of derivation of sequence models and differences in the hidden Markov model architectures.

To assess how effective the filters were, we evaluated MetaGeneTack’s performance produced with various combinations of filters and compared with performance of FragGeneScan on fragment sets with insertion FS (Fig. 1A) and sets with deletion FS (Fig. 1B). Here we show results for 600 nt-long sequences with 20% fragments containing frameshifts. Without filters, the *Sn* of MetaGeneTack was close to FragGeneScan while the *Sp* was >10% higher in both cases. With the filters, the average *Sn* and *Sp* of MetaGeneTack increased by ∼5 percentage points. Similar results were observed when an FS prediction was reported as a true positive if located within 10 nt from the simulated FS (data not shown). The distribution of the distance between predicted FS positions and true FS positions is shown in Supplementary Figure S1. The standard deviation is 10.3 and 12.6 for MetaGeneTack and FragGeneScan, respectively.

## 4 CONCLUSION

The new software program, MetaGeneTack, addresses the challenging question of predicting frameshifts in protein-coding regions of metagenomic sequences without extrinsic knowledge. An advantage of ab initio approach is the ability to detect frameshifts in genes of orphan proteins that do not have known homologs. We have shown that the accuracy of MetaGeneTack is comparable or better than the accuracy of the ab inito gene prediction tool FragGeneScan. Most of the frameshifts predicted by MetaGeneTack are supposed to result from sequencing errors; still like GeneTack, the program is also able to detect frameshifts caused due to indel mutations and ones related to recoding (programmed frameshifts involved in gene regulation). MetaGeneTack could be integrated into pipelines of metagenomic sequence annotation.

## Supplementary Material

Supplementary Data
