# Supplementary material for: MetaGeneTack: ab initio detection of frameshifts in metagenomic sequences
Source: Bioinformatics. 2012 Nov 4;29(1):114–6. doi: 10.1093/bioinformatics/bts636 (PMC3530910; doi:10.1093/bioinformatics/bts636)
Supplement: Supplementary Data [file supp_29_1_114__index.html]

MetaGeneTack: Ab Initio Detection of Frameshifts in Metagenomic Sequences — MetaGeneTack: ab initio detection of frameshifts in metagenomic sequences — Supplementary Data 

# MetaGeneTack: ab initio detection of frameshifts in metagenomic sequences

## Supplementary Data

files

**Files in this Data Supplement:**

- Supplementary Data - pdf file
- Supplementary Data - tex file
