# Supplementary material for: MetaGeneTack: ab initio detection of frameshifts in metagenomic sequences
Source: Bioinformatics. 2012 Nov 4;29(1):114–6. doi: 10.1093/bioinformatics/bts636 (PMC3530910; doi:10.1093/bioinformatics/bts636)
Supplement: Supplementary Data [file supp_bts636_Supplementary_MetaGeneTack.pdf]

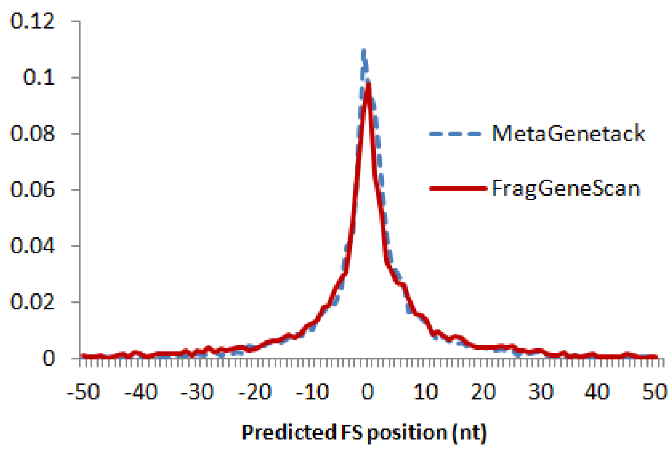

Figure S1: Distributions of the distance between predicted FS positions and true FS positions for 400nt, 600nt, and 800nt fragments with simulated frameshifts (Table 1). Deviations longer than 50nt are not shown. The standard deviations of the distributions are 10.3 and 12.6 for MetaGeneTack and FragGeneScan, respectively.

Table S1: Frameshift prediction accuracy for 400nt fragments from 18 prokaryotic genomes (with 20% of fragments containing frameshifts). Avg denotes the average of Sn and Sp. In general, MetaGeneTack reaches higher accuracy for bacteria than archaea (indicated by stars in Table S1). Interestingly, Sn of MetaGeneTack in frameshift detection is higher in high GC and low GC genomes than in middle GC genomes.

			**FragGeneScan**			**MetaGeneTack**		
	Sequence ID	GC%	Sn	Sp	Avg	Sn	Sp	Avg
Methanosphaera stadtmanae ∗	NC_007681	28	87.3	62.0	74.6	74.9	83.8	79.3
Campylobacter jejuni	NC_002163	31	88.6	50.6	69.6	84.3	67.3	75.8
Staphylococcus aureus Mu50	NC_002758	33	88.6	51.9	70.2	76.6	83.1	79.8
Picrophilus torridus DSM 9790 ∗	NC_005877	36	52.0	24.5	38.3	74.4	70.8	72.6
Streptococcus pyogenes M1 GAS	NC_002737	39	87.6	47.6	67.6	70.3	76.9	73.6
Pasteurella multocida	NC_002663	40	85.3	57.0	71.1	70.8	75.5	73.1
Bacillus subtilis	NC_000964	44	78.3	39.6	58.9	64.3	71.9	68.1
Thermotoga maritima	NC_000853	46	60.8	28.7	44.7	66.8	61.5	64.1
Archaeoglobus fulgidus ∗	NC_000917	49	63.9	24.9	44.4	80.4	58.8	69.6
Escherichia coli K12	NC_000913	51	83.6	42.6	63.1	76.8	71.1	73.9
Pyrobaculum aerophilum ∗	NC_003364	51	65.0	27.8	46.4	60.1	55.1	57.6
Salmonella typhimurium LT2	NC_003197	52	85.6	43.9	64.8	75.4	70.7	73.0
Thermococcus kodakaraensis KOD1 ∗	NC_006624	52	69.1	27.8	48.5	79.4	59.7	69.5
Methanopyrus kandleri ∗	NC_003551	61	81.3	36.1	58.7	68.3	60.4	64.3
Caulobacter crescentus	NC_002696	67	94.6	59.7	77.2	83.8	73.3	78.5
Ralstonia solanacearum	NC_003296	67	94.3	48.8	71.5	86.6	70.7	78.7
Clavibacter michiganensis	NC_010407	73	95.0	47.3	71.2	83.9	70.9	77.4
Anaeromyxobacter dehalogenans	NC_007760	75	96.4	57.5	76.9	87.1	82.4	84.8
**Average**			81.0	43.2	62.1	75.8	70.2	73.0
